# Supplementary figures and images for: Lactate induces synapse-specific potentiation on CA3 pyramidal cells of rat hippocampus
Source: PLoS One. 2020 Nov 12;15(11):e0242309. doi: 10.1371/journal.pone.0242309 (PMC7660554; doi:10.1371/journal.pone.0242309)

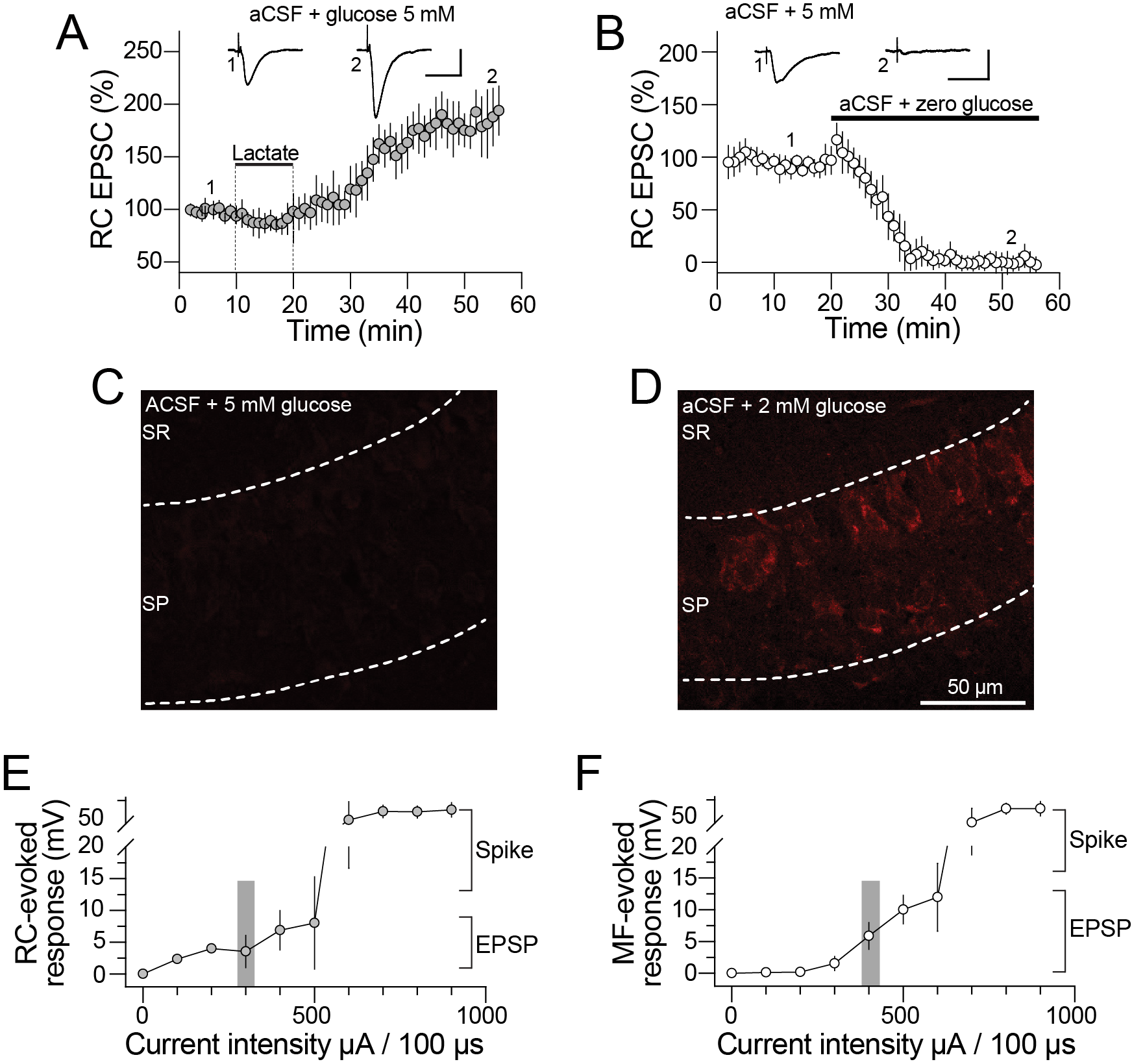

Supplement: S1 Fig — A) Average time-course graph showing the effects of lactate 2 mM on the RC EPSC acquired in an artificial cerebrospinal fluid (aCSF) containing glucose 5 mM. Under this condition, the passive and active electrophysiologic properties of CA3 PC were similar to the recorded in the standard aCSF containing glucose 10 mM. The synaptic potentiation triggered by lactate (2 mM, 10 min) was similar to the potentiation reported in Fig 1D. Inset, representative RC EPSC obtained in control conditions (1) and 30 min after lactate washout (2). B) Average time-course graph showing the effects of removing glucose on the normalized RC EPSC amplitude acquired in an aCSF containing glucose 5 mM. As previously reported (Schurr et al., 1988; Tecuatl et al., 2018), removing glucose irreversibly suppressed the RC EPSC. Upper inset, representative RC EPSC obtained in control conditions (1) and 30 min after removing glucose (2). The suppression of the evoked responses on CA3 PC was accompanied by propidium iodide accumulation, indicating reduced cell viability. C-D) Higher magnifications of confocal microphotographs showing the PI uptake in slices maintained in aCSF containing glucose 5 mM or low glucose concentration. PI accumulation (red staining) was observed in slices containing as low as 2 mM of glucose. The details for propidium iodide uptake and fluorescence microscopy on acute hippocampal slice has been previously published by our group (Tecuatl et al., 2018). E-F) Scatter plots of current intensity vs. EPSP amplitude acquired in current-clamp mode at RMP of CA3 PC. The input-output curves were used to determine a stimulus that caused 30–40% of the subthreshold response to avoid saturation (spike generation) following repetitive stimulation (20 stimuli at 20 or 40 Hz at 10-s intervals; See Fig 8). RC synapses required less current injection to generate spikes, whereas MF stimulation triggered larger synaptic responses before spike onset. The gray areas on the curve represent the inj [file pone.0242309.s001.tif]

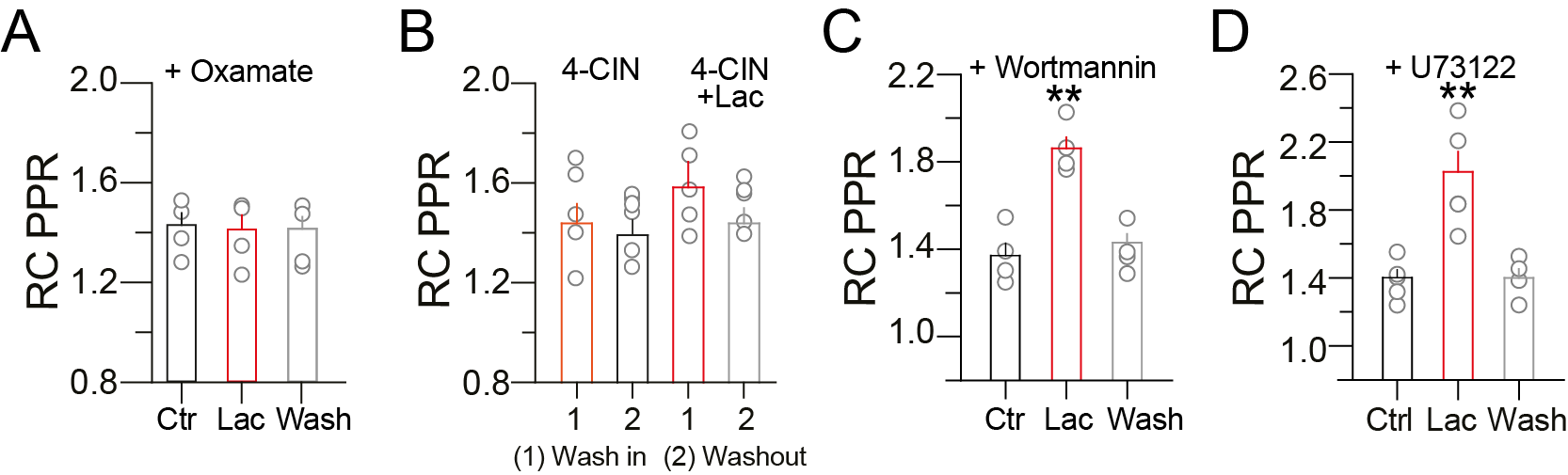

Supplement: S2 Fig — A) Extracellular perfusion of the non-competitive inhibitor of the lactate dehydrogenase, oxamate (10 mM, 20 min), prevented the increase in the PPR during lactate perfusion. B) Perfusion of the monocarboxylate transporter-2 blocker, 4-CIN (0.5 mM) did not alter the PPR at the RC synapse of CA3 PC but prevented its increase during lactate perfusion. C) Somatic loading with the IP3-K signaling cascade blocker, wortmannin (10 μM), does not interfere with the modulation of PPR by lactate. Likewise, D) Somatic loading with the inhibitor of the Protein lipase C (PLC), U73122 (10 μM) does not interfere with the increase in PPR exerted by perfusion of lactate. For all the bars (Mean ± SEM), each symbol represents an independent experiment. (TIF) [file pone.0242309.s002.tif]
